# Vacuum-Assisted Synthesis of Solid-State Fluorescent Carbon Quantum Dots for Color Conversion LEDs

**DOI:** 10.1021/acsomega.5c01047

**Published:** 2025-04-11

**Authors:** Hikmet Altintas, Kevser Sahin Tiras

**Affiliations:** Department of Physics, Faculty of Science, Erciyes University, Kayseri 38030, Turkey

## Abstract

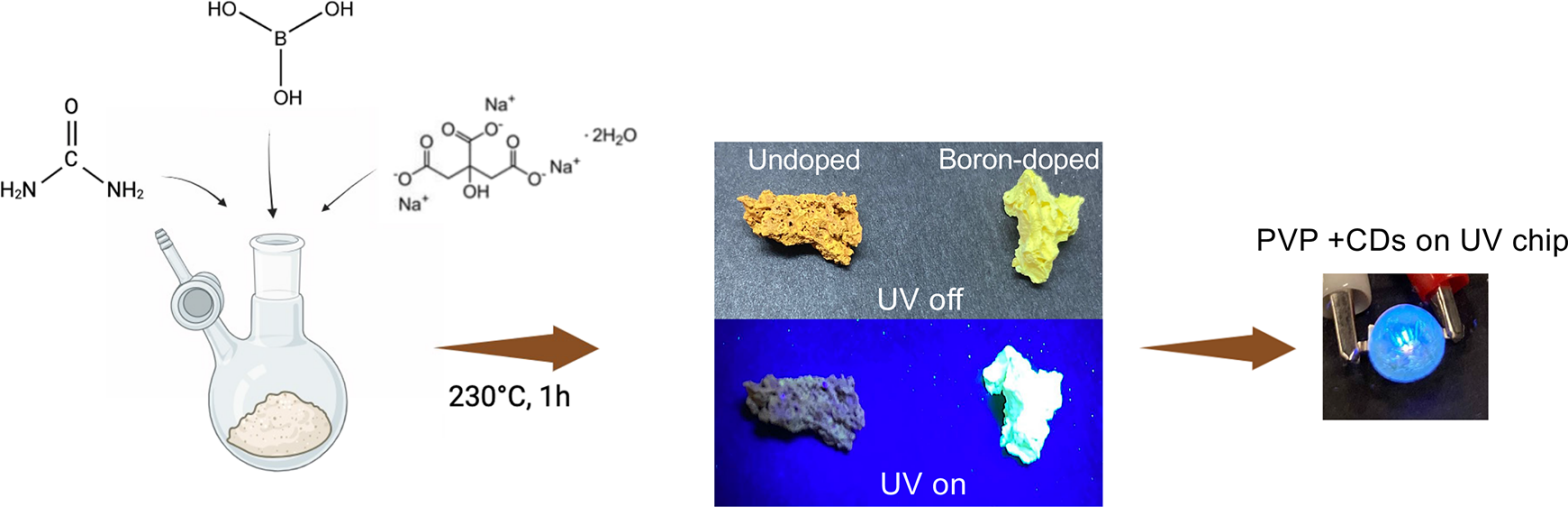

Carbon dots (CDs) are one of the most promising nanomaterials with tunable optical properties and very good biocompatibility, opening wide perspectives in sensing, imaging, and optoelectronic applications. However, fluorescent CDs in powder form that are both adjustable and effective remain a significant issue. Here, we describe a simple, quick, and scalable process for creating solid-state green-emissive CDs using urea and trisodium citrate dihydrate with boric acid as a matrix and no dispersion solvent. The process employs a vacuum-assisted synthesis method, which enhances the molecular interaction between the precursors and ensures uniform dispersion, significantly improving the quality and stability of the final product. CDs embedded in a boric acid matrix (B-CDs) exhibit a photoluminescence quantum yield (PLQY) with a nearly 18% decrease when transitioning from aqueous solution to solid-state films. In contrast, CDs without the boric acid matrix display a significantly lower PLQY in aqueous form and no luminescence in the solid state, highlighting the enhancing effect of the boric acid matrix. By effectively reducing the aggregation-induced quenching, the boric acid matrix’s spatial confinement is thought to cause this rise in fluorescence. The resulting B-CD powders exhibit adjustable CIE coordinates and have been used to fabricate color-conversion light-emitting devices on UV chips. The current study presents a viable and scalable approach to solid-state fluorescent CDs that are very stable and efficient. These CDs will find extensive potential use in luminescent devices based on CDs, ranging from flexible lighting systems to color conversion, opening a new era of possibilities.

## Introduction

Carbon dots (CDs) have recently received much attention due to their specific optical features, extremely high photoluminescence, excellent stability, and biocompatibility. These nanoscale carbon materials usually fall within the 5–20 nm range and exhibit size-dependent fluorescence. They were widely used in bioimaging, sensing, and optoelectronics.^1,2^ Solid-state emissive CDs have numerous benefits over conventional light-emitting materials such as rare earth phosphors and semiconductor quantum dots (SQDs).^3−7^ CDs have limited applicability due to significant photoluminescence (PL) quenching in the solid state, also known as aggregation-caused quenching (ACQ).^8,9^ Therefore, the superior photoluminescent capabilities of CDs in a dispersed state are typically unsuitable in solid-state environments. To overcome this challenge, Zhou et al. developed a method to prepare strongly luminescent CDs@silica composite gels that prevent aggregation and achieve high quantum yields.^10^ Kwak et al. synthesized polymer CDs with consistent emission color and high quantum yield in both the solution and the solid state.^11^ Zhu et al. modulated the emissive states of colloidal CDs and dispersed them in a polyvinylpyrrolidone (PVP) matrix, which gave a white light quantum yield of 38.7% in the solid-state films.^12^ Wang et al. discussed some strategies for the preparation of fluorescent solid-state CD materials, including adjusting the optical properties and exploring their applications in optoelectronics, biology, and sensing.^13^ These results represent significant advances in conquering solid-state photoluminescence quenching of CDs, paving the way for practical applications.

Most synthesis techniques for CDs via a solid-state reaction involve the thermal treatment of carbon-rich precursors, which undergo carbonization and further structural rearrangements into quantum dots.^8^ This approach improves the quantum yield of the obtained CDs and allows for effective dopant or functional group introduction, which can further tune their optical properties. Controlling the emission wavelength by doping and size manipulation opens exciting perspectives for color conversion applications, which are essential in areas such as LED technology and display systems.^14^

Recent reports have highlighted the potential of CDs in color conversion applications.^15−17^ CDs can serve as efficient phosphors for converting blue light to assorted colors, enhancing the color rendering index of white LEDs.^18^ Encapsulating CDs in LED matrices can improve efficiency and broaden the spectral output, enabling the generation of tunable white light with superior color quality.^19^ Furthermore, the solid-state form of CDs facilitates their integration into commercial products, offering a promising alternative to traditional phosphors.^20^

Vacuum heating techniques for creating highly luminous CDs with improved solid-state performance have been developed.^12^ In this method, the foam structure in which the growth of CDs is space-confined enables the enhancement in photoluminescence quantum yield (PLQY) and reduction of aggregation-induced quenching.^8,9^ The vacuum heating method, in conjunction with metal cations or boric acid, promotes the formation of a porous matrix, thereby facilitating the in situ synthesis of CDs.^21^ This allows controllable synthesis, yielding tunable optical properties of the CDs for various applications such as in luminescent solar concentrators and anticounterfeiting.^9^ Moreover, heating under a vacuum allows the synthesis of multicolored room temperature phosphorescence emissions with extended lifetimes that are promising candidates for security codes and information encryption systems.^22^ In a related study, this effect was achieved through the formation of luminescent boron-doped CD centers within a rigid polycrystalline B_2_O_3_ glassy matrix, which stabilizes triplet excited states and enables highly tunable, long-lasting phosphorescence across a full-color spectrum.^23^

Generally, before synthesis, CD precursors have been dissolved in a solvent to achieve a homogeneous mixture. However, in this present study, no liquid dispersion medium was used, and all the precursors were directly mixed and subjected to vacuum heating for the synthesis process. The solid-state reaction method was used to synthesize CDs, and their properties were thoroughly studied for potential applications in color conversion technologies. Boron-doped CDs (B-CDs) were synthesized via a vacuum heating method using trisodium citrate dihydrate, urea, and boric acid, following a procedure akin to previously reported methods in the literature.^8,20^ This method offers a cleaner, more efficient, and environmentally friendly alternative to traditional solvent-based synthesis. Additionally, other studies have also explored the use of boric acid and trisodium citrate dihydrate in similar processes to enhance the synthesis of CDs.^24−28^ For comparative purposes, CDs without boric acid (BA) were synthesized under identical reaction conditions, including the reaction temperature, time, and the molar ratio of urea to trisodium citrate dihydrate. Color-conversion LEDs were fabricated by dispersing the aqueous form of B-CDs into PVP. The synthesized CDs and B-CDs exhibited luminescent properties in aqueous solutions; however, only the B-CDs retained luminescence in solid form. The B-CDs demonstrated a PLQY of 22% in aqueous form and 18% in film form.

## Experimental Section

### Materials

Trisodium citrate dihydrate (Sigma-Aldrich), urea (99.0% Isolab), polyvinylpyrrolidone (PVP), and boric acid (Emsure) were used as received.

### Synthesis

**Scheme 1:**
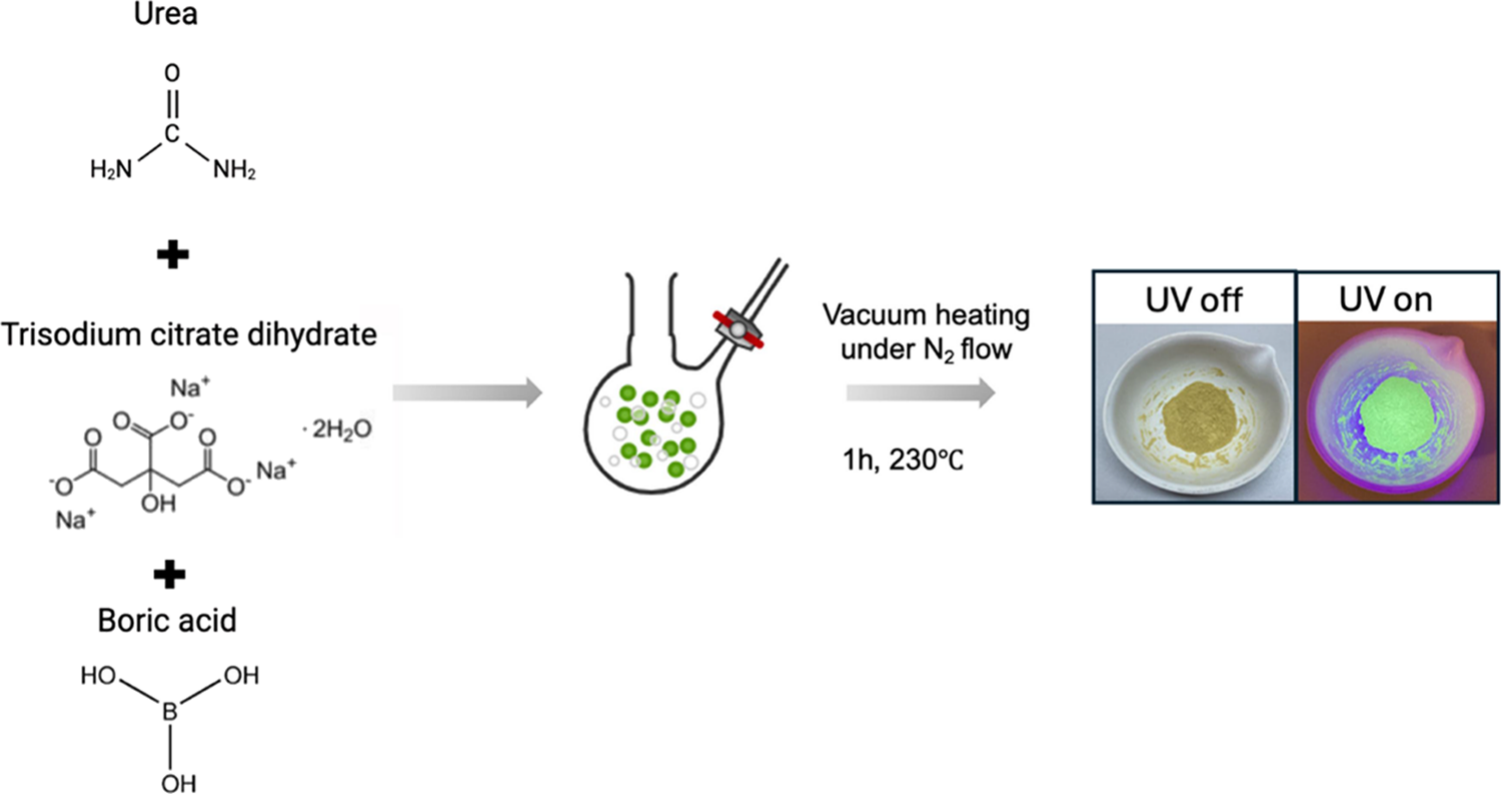
Schematic of Synthesis of Boron-Doped CDs

The vacuum heating synthesis was conducted using a systematic approach.^8,17^ For the synthesis of B-CDs, a three-neck flask was charged with 1.5 g of trisodium citrate dihydrate, 0.33 g of urea, and 1.3 g of boric acid. To evaluate the effect of boric acid, a control batch of CDs was synthesized with 1.5 g of trisodium citrate dihydrate and 0.33 g of urea. Both syntheses followed an identical procedure, which is outlined as follows: A mechanical oil pump was used to establish a vacuum, while the reaction mixture was gradually heated to 50 °C. Subsequently, the temperature was raised to 230 °C under a continuous flow of nitrogen gas and maintained for 1 h. This thermal treatment facilitated the conversion of the precursor materials into a spherical, porous foam structure. After cooling to room temperature, the resulting inflated foams were easily crushed into fine powders using an agate mortar. Schematic of the synthesis of B-CDs is shown in Scheme 1. The final product exhibited complete solubility in water.

### Color Conversion LED Fabrication

CDs’ concentration-dependent PL properties in the dispersion phase and their green light emission in the solid state make them promising candidates for various solid-state lighting applications, including light-emitting thin films and color conversion devices. PVP is a water-soluble polymer that, upon mixing, facilitates better dispersion of the CDs in water or any polar solvent, maintaining their distribution and enhancing their interaction with light in LEDs. B-CDs and PVP mass ratios were combined to fabricate solid-state light-emitting thin films to investigate the concentration-dependent PL behavior. The CDs function as color-converting dopants in this system, while PVP is the host matrix. Different concentrations of powder B-CDs (6, 12, 24, and 36 mg) were mixed with 1 mL of a PVP solution (50 mg/mL) for color conversion applications. Given that the synthesized CDs are fully soluble in water, a water-soluble polymer was selected as the host material for the film formulation. A 100 μL blend was deposited onto a Petri dish and allowed to dry at room temperature for 24 h. The resulting B-CDs-PVP films were then evaluated for color conversion efficiency using a 395 nm UV LED chip as the excitation source. The color conversion properties of the films were assessed based on their emission spectra and fluorescence characteristics under UV irradiation.

### Characterization

The X-ray diffraction (XRD) pattern was collected via the Bruker D8 Discover. The Fourier transform infrared (FTIR) spectroscopy was performed using the FT-IR spectroscopy model NICOLET 6700 made by Thermo Scientific. The EDX elemental analysis was performed by Thermo Fisher Scientific, which was equipped with an ELECT plus detector and Gemini 300 microscope. The surface morphology were examined using scanning electron microscopy (SEM) with a ZEISS GEMINI 500 instrument. A FEI TALOS F200S transmission electron microscope was used at an operating voltage of 200 kV to capture a transmission electron microscopy (TEM) image. The X-ray photoelectron emission spectrum (XPS) was acquired via a PHI 5000 Versa Probe X-ray spectrometer. The optical spectroscopies were performed via UV–visible (UV–vis), PL/PL excitation (PLE), and time-resolved PL (TRPL) spectroscopies and measurements of the PLQY. Data were recorded using a Thermo Genesys 10S spectrometer, a Cary Eclipse, a PicoQuant Fluo Time 200 time-correlated single photon counting system (TCSPC), and a Quantaurus-QY instrument made by Hamamatsu Company, respectively. Electroluminescence (EL) spectra were measured by Ocean Optics HR 4000 CG-UV-NIR series high-resolution fiber optic spectrometer, which couples a linear charge-coupled device (CCD)-array detector ranging from 200 to 1100 nm.

## Results and Discussion

**Figure 1:**
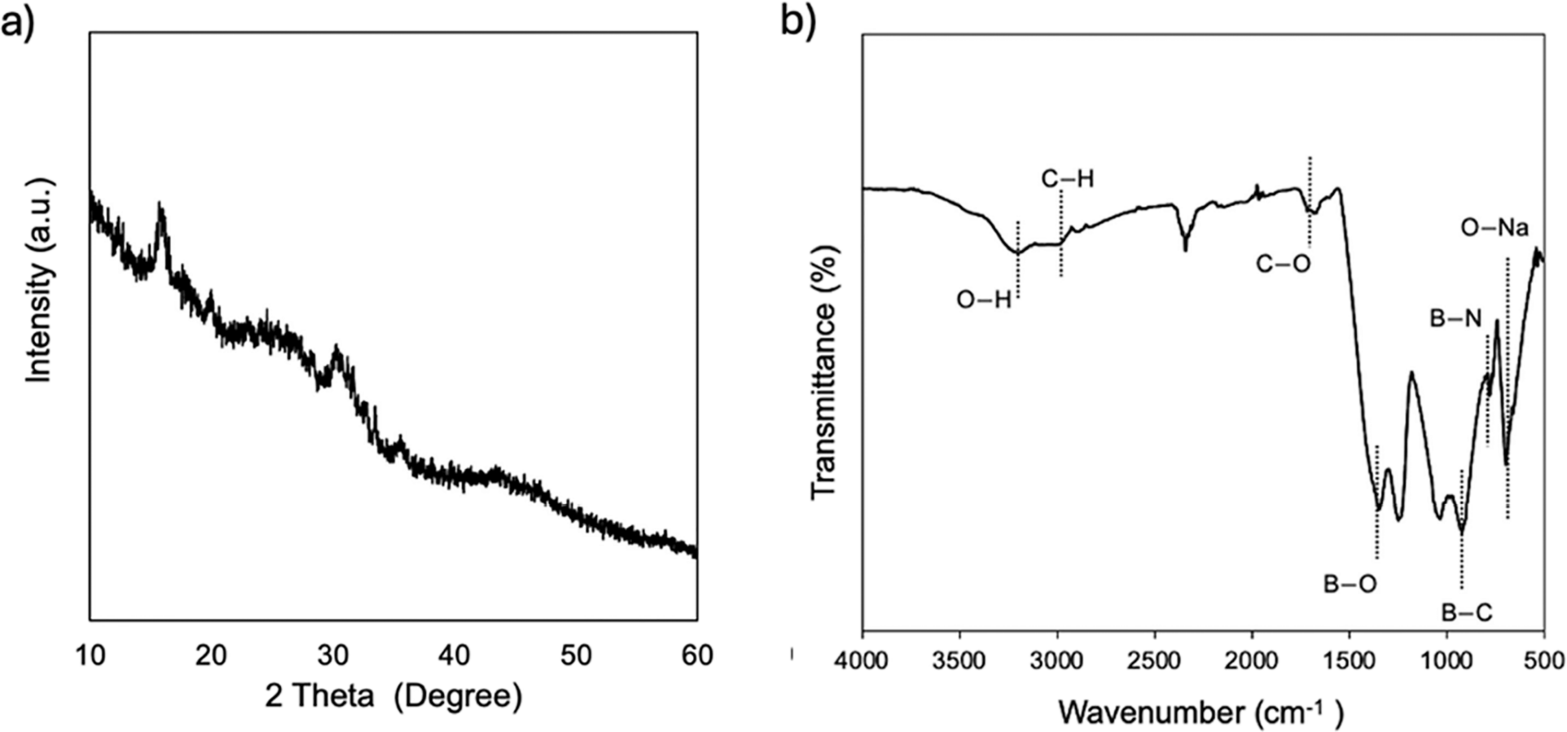
(a) XRD pattern and (b) FTIR spectra of B-CDs.

The powder and aqueous forms of CDs and B-CDs were characterized using optical and elemental analysis techniques. Materials based on graphene and CDs have unique optical and structural characteristics. Even though carbon-based QDs are frequently luminous, their crystallinity is typically weak, exhibiting an amorphous carbonate and a graphite-like core. Because of their disordered structure and absence of long-range crystalline order, these materials usually exhibit broad peaks in their X-ray diffraction (XRD) patterns.^29,30^ As shown in Figure 1a, the broad diffraction peak around 2θ values of 15–30° is commonly associated with the (002) plane, indicating a graphitic structure and sp^2^ hybridization.^20,31^

To explore the chemical structure of the composites, Fourier transform infrared (FT-IR) and X-ray photoelectron spectroscopy (XPS) were further applied. The FT-IR spectra of powder B-CDs are shown in Figure 1b. The distinctive band observed at 696 cm^–1^ is attributed to the wagging vibrations of O–Na.^32^ Three characteristic peaks of B–N, B–C, and B–O at 779, 922, and 1345 cm^–1^, respectively, indicate the creation of covalent bonds between CDs and the boric acid matrix, mainly shown by the presence of B–C.^33^ The C–O band is visible at the peak at 1684 cm^–1^.^32^ The stretching vibration of O–H and C–H is responsible for the peaks at 3183 and 2989 cm^–1^.^18^

**Figure 2:**
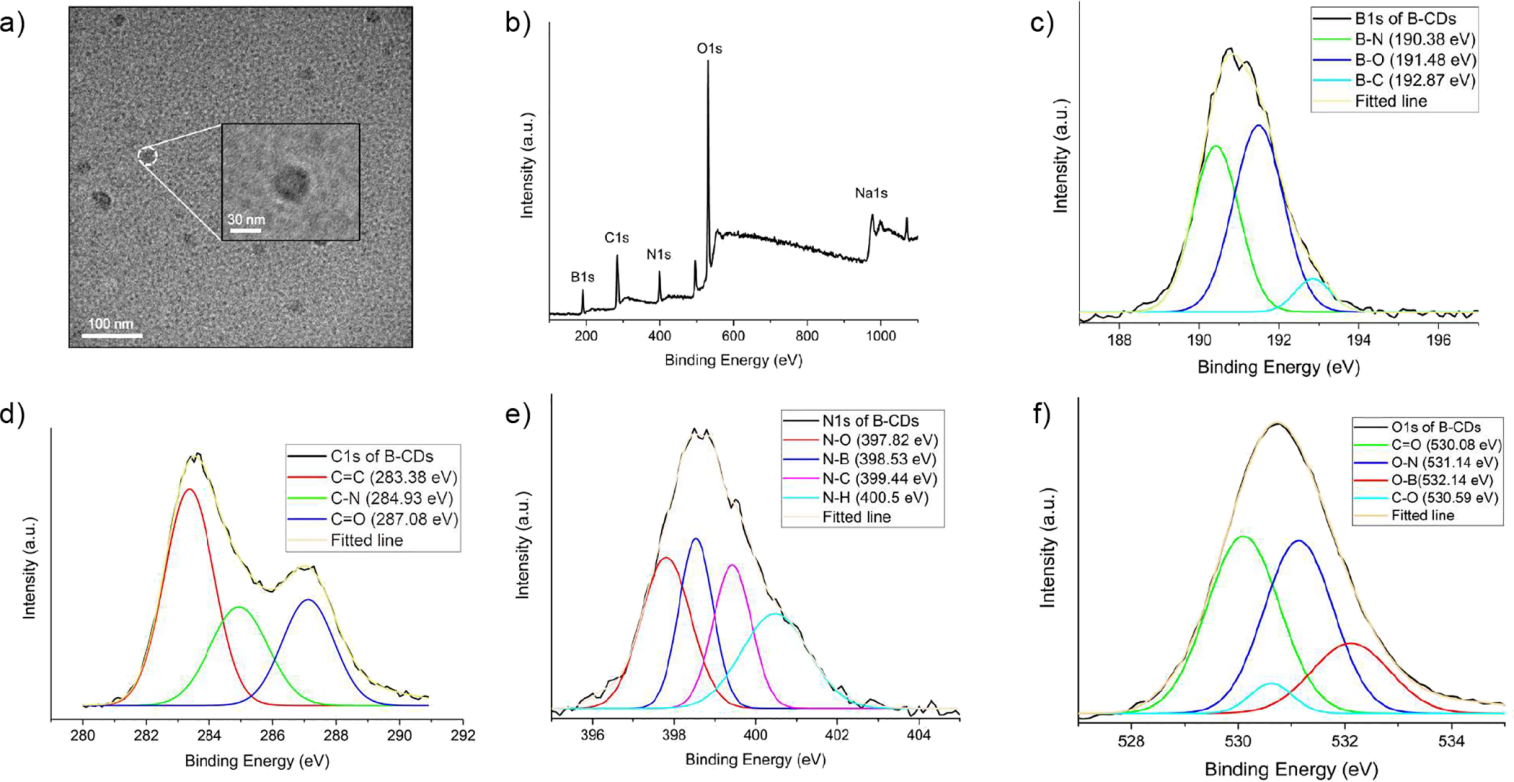
(a) TEM image of B-CDs, (b) XPS survey of B-CDs, high-resolution XPS survey of (c) B 1s, (d) C 1s, (e) N 1s, and (f) O 1s of B-CDs.

The existence of elements with varying element ratios, such as C (25.2%), N (32.57%), O (27.38%), and Na (14.85%) for CDs and C (10.11%), N (8.45%), O (57.85%), Na (3.36), and B (20.22%) for B-CDs, was also demonstrated via EDX analysis in Figure S1a,b, respectively. The solid-state foam structure created by the vacuum heating method may limit the precursors’ diffusion, resulting in the space-confined growth of CDs confirmed by SEM images of both CDs and B-CDs in Figure S2a,b, respectively.^8,34^ The transmission electron microscopy (TEM) image in Figure 2a reveals that the B-CDs have an average size of approximately 30 nm. To further investigate the composition of B-CDs, XPS analysis was performed, as shown in Figure 2b. The full XPS spectrum reveals that the B-CDs are primarily composed of Na, N, B, C, and O, with atomic ratios of 2.8, 8.1, 16.7, 32.1, and 40.3%, respectively. Figure 2c presents the high-resolution B 1s spectrum, where three peaks at 190.38, 191.48, and 192.87 eV are assigned to B–N, B–O, and B–C bonds, respectively.^24^ The C 1s spectrum in Figure 2d displays peaks at 283.38, 284.93, and 287.08 eV, corresponding to C=C, C–N, and C=O bonds.^27^ Additionally, the high-resolution N 1s and O 1s spectra shown in Figure 2e,f, with peaks at 398.53 and 532.14 eV, respectively, correspond to N–B and O–B, further confirming the formation of B–N–O bonds.^24^

**Figure 3:**
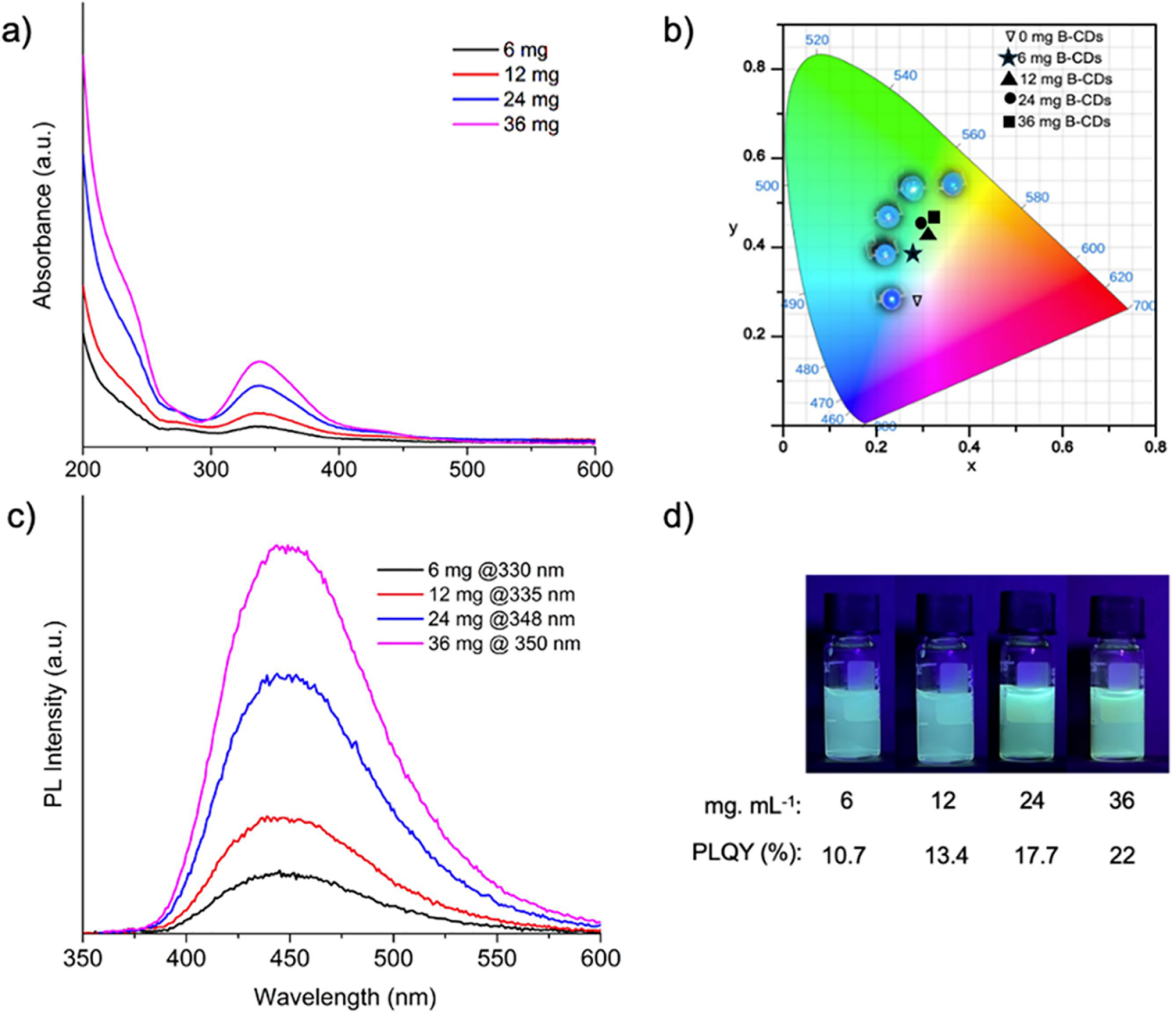
(a) Absorbance spectra of aqueous B-CDs at various concentrations, (b) CIE color coordinates of B-CDs-PVP films, with corresponding photographs of the films illuminated on a UV chip, (c) PL spectra of aqueous B-CDs at various concentrations at different excitation wavelength, (d) Photographs of the 395 nm UV irradiated B-CDs solution with B-CD concentrations and corresponding PLQY values. The images are arranged from left to right, corresponding to increasing B-CD concentrations.

The optical characteristics of B-CDs at different concentration rates, a primary physical characteristic of a nanomaterial that influences their use in LEDs, were evaluated. UV–vis spectroscopy of aqueous B-CDs at different concentration rates representing characteristic absorption between 300 and 400 nm, as seen in Figure 3a. A 395 nm UV chip was selected as the excitation source and combined with a mixture of B-CDs-PVP films to fabricate color conversion LEDs. The operating parameters for the chip were 3.07 V and 2 mA. The CIE coordinates of color conversion LEDs fabricated using a mixture of B-CDs and PVP are presented in Figure 3b. The B-CD doping levels in the PVP solution vary from 0 to 36 mg, with specific concentrations of 0, 6, 12, 24, and 36 mg. The corresponding CIE coordinates for these samples are (0.28, 0.27), (0.28, 0.38), (0.31, 0.42), (0.29, 0.45), and (0.32, 0.47), respectively. As the doping level increases, the CIE coordinates shift toward higher values on the chromaticity diagram, indicating a noticeable change in the color emission of the LEDs. This variation suggests that the doping concentration of CDs plays a significant role in tuning the color characteristics of the LEDs, providing a potential approach for color-tuning applications in optoelectronic devices.

The PL spectra of aqueous B-CDs at various concentration levels, measured at different excitation wavelengths, are shown in Figure 3c. The emission spectra consistently exhibit a dominant peak around 445 nm, which becomes more pronounced with increasing concentration. As observed, the PL intensity increases with B-CD concentration, indicating a concentration-dependent enhancement in luminescent emission. This trend suggests that higher concentrations of B-CDs lead to stronger PL signals, which may be attributed to the increased number of emitting sites within the solution. Photographs of the 395 nm UV-irradiated aqueous B-CDs at various concentrations, along with the corresponding PLQY values, are shown in Figure 3d. The PLQY of aqueous B-CDs was calculated using the comparative method, with diphenyl anthracene (PL peaks at 406 and 427 nm) as the reference dye. As the concentration increases, a noticeable enhancement in PLQY values is observed. This progression highlights the concentration-dependent behavior of B-CDs, with higher concentrations leading to more intense luminescent emissions.

**Figure 4:**
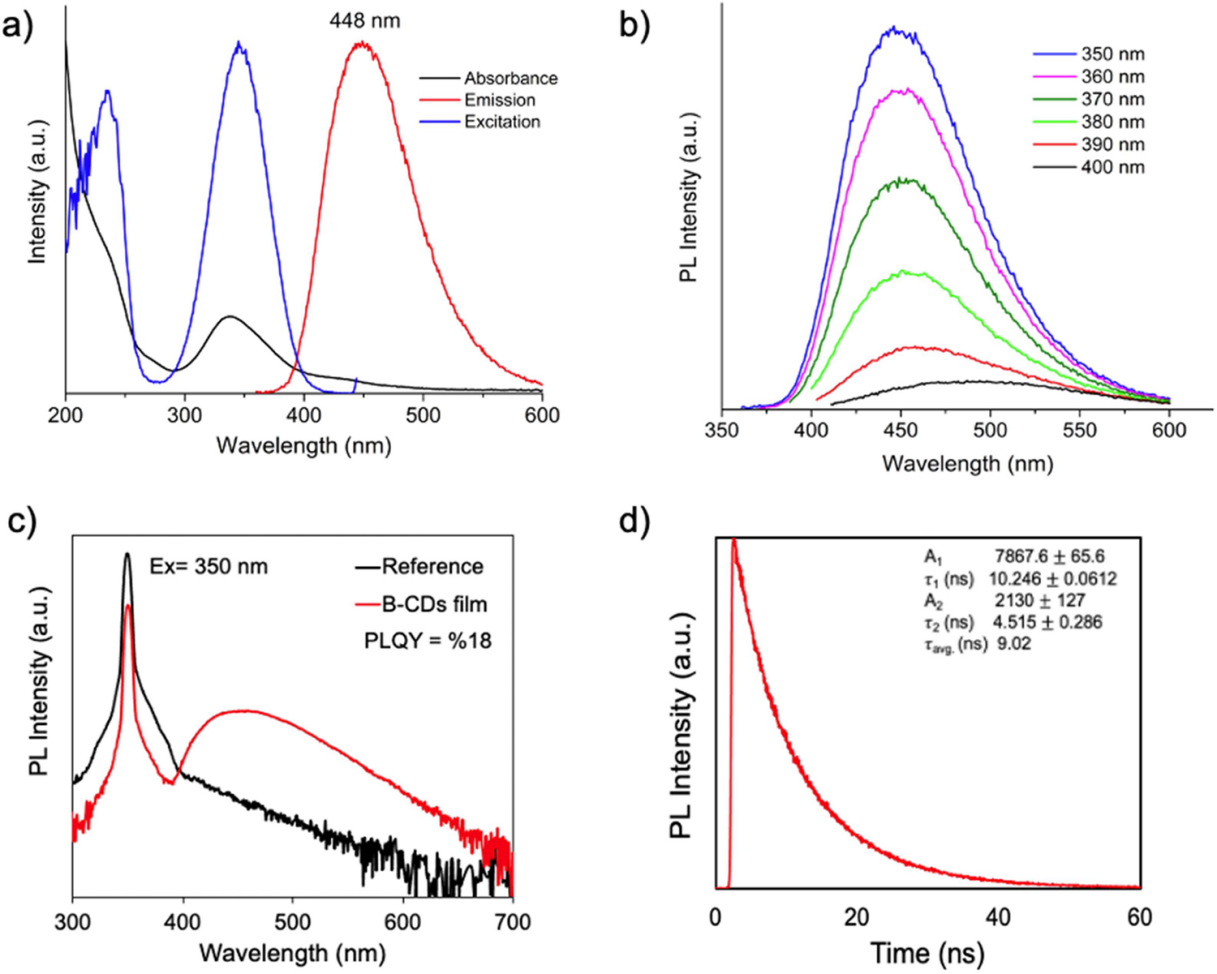
(a) UV–vis, PLE (λ_ems_ = 448 nm), and PL (λ_exc_ = 350 nm) spectra, (b) excitation-dependent PL spectrum recording from 350 to 400 nm, (c) absolute PLQY measurement for B-CDs mixed PVP film, (d) logarithmic TRPL profile of the corresponding B-CDs obtained at a wavelength of 448 nm (the inset shows the fitting parameters) of aqueous B-CDs.

We further evaluated the PL, PLE, and UV–vis spectra of aqueous B-CDs at 36 mg mL^–1^ concentration, as shown in Figure 4a. The UV–vis absorbance spectra of B-CDs display a peak at 234 nm, which corresponds to the π–π* transition linked to the sp^2^ hybridization of the carbon–carbon double bonds (C=C). Additionally, a strong peak was observed around 336 nm, attributed to the n−π* transition associated with the sp^3^ hybridization, suggesting the presence of bonds between carbon atoms and functional groups containing nitrogen and oxygen.^35,36^ Strong excitation signals are also visible in the PLE spectra at 345 and 235 nm, consistent with the UV–vis result. A broad fwhm at 85 nm and a strong signal at 448 nm was observed in the PL spectra, which were taken at an excitation wavelength of 350 nm to determine the emission characteristics, yielding a PLQY of 22%. The PL peak position reveals a significant Stokes shift associated with the absorption peak of nearly 100 nm, confirming the crucial function of localized midgap states in optical recombination.

As shown in Figure 4b, the position of the PL spectrum remains constant across different excitation wavelengths, indicating the presence of homogeneous radiative centers within the B-CDs. This wavelength-independent emission behavior suggests that the radiative recombination process is uniform throughout the material, further supporting the potential for consistent optical performance in various applications.

There are multiple types of fluorescent centers with varying energy levels in a B-CD, and this can be due to the carbon core, surface functional groups, or quantum confinement effects. The excitation-dependent PL behavior and broad PL spectrum of B-CDs in Figure 4b arise from the coexistence of numerous photoluminescent centers with a wide range of energy levels, where transitions between these centers and their relative contributions depend on the excitation wavelength.^1^ Given the PLE’s extended peak location, it displayed the highest emission intensity at excitation wavelengths of 350 nm, as expected.

To validate these results and assess any significant changes in the film form of the B-CDs-PVP blend, the PLQY was re-evaluated using an integrated measurement system in Figure 4c, which yielded a value of 18% at an excitation wavelength of 350 nm. The PLQY values for both aqueous B-CDs and the B-CDs-PVP film blend are in close agreement, which is noteworthy as CDs typically tend to aggregate in the solid state, suggesting stable photoluminescent properties in both forms. Time-resolved photoluminescence (TRPL) measurements were carried out to explore the energy levels that control the recombination process and to determine the average emission lifetime. The TRPL data in Figure 4d for the aqueous B-CDs exhibited a biexponential decay profile, which can be attributed to midgap radiative recombination centers. The measured photoluminescence lifetime was 9.02 ns, as summarized in the inset of Figure 4d, and the decay kinetics were well-described by a two-component exponential model, suggesting the involvement of multiple recombination pathways.^37,38^ This biexponential behavior provides an essential insight into the complex charge carrier recombination dynamics within the CDs.^39^ Additionally, TRPL data for undoped CDs are shown in Figure S3, where the measured photoluminescence lifetime is 5.49 ns, with a summary provided in the inset. The three exponential components of the CDs are typically associated with three distinct recombination pathways, indicating the presence of multiple emissive centers.^24,37^ These results suggest that boron doping in CDs enhances the photoluminescence lifetime, particularly in aqueous environments.

**Figure 5:**
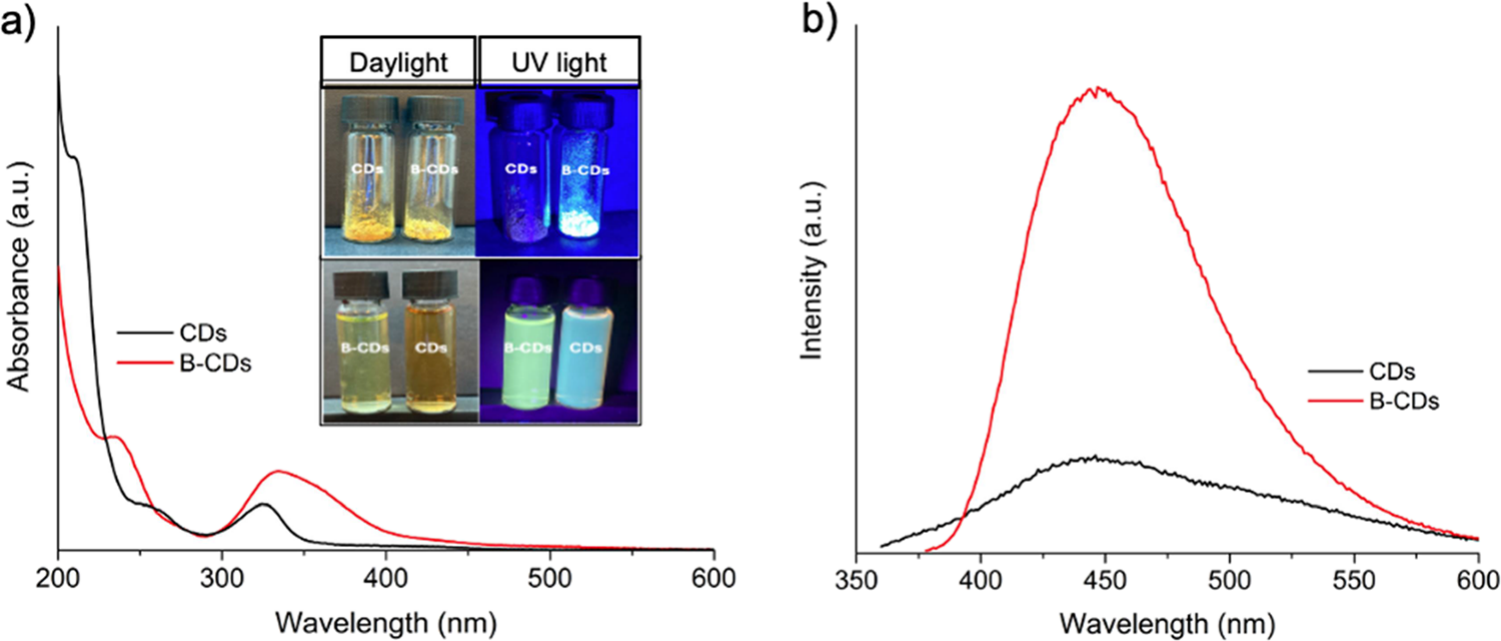
(a) Absorbance spectra of CDs and B-CDs in aqueous form (inset: photographs of the solid (upper) and aqueous (lower) forms for both CDs and B-CDs under daylight and UV light), and (b) PL intensities of CDs and B-CDs in aqueous form.

As previously mentioned, both CDs and B-CDs are water-soluble; however, solid-state luminescence is observed exclusively in B-CDs, with no fluorescence detected from the unmodified CDs in their solid form. The actual photographs of the solid and aqueous states for both types of dots under daylight and UV light are provided as an inset in Figure 5a. A comparative analysis was performed in an aqueous solution, and Figure 5a,b present the differences in absorbance and PL intensities between the two types of dots in water at the same concentrations. The PLQY values were determined through a comparative method, yielding values of 9% for CDs and 22% for B-CDs when excited at 350 nm. This analysis leads to the conclusion that boron doping induces luminescence in CDs in their solid state and enhances their optical properties in aqueous solutions. B-CDs’ absorbance and PL intensities are significantly improved compared to undoped CDs. Therefore, boron doping plays a crucial role in enhancing the optical performance of CDs, both in solid and aqueous forms.

**Figure 6:**
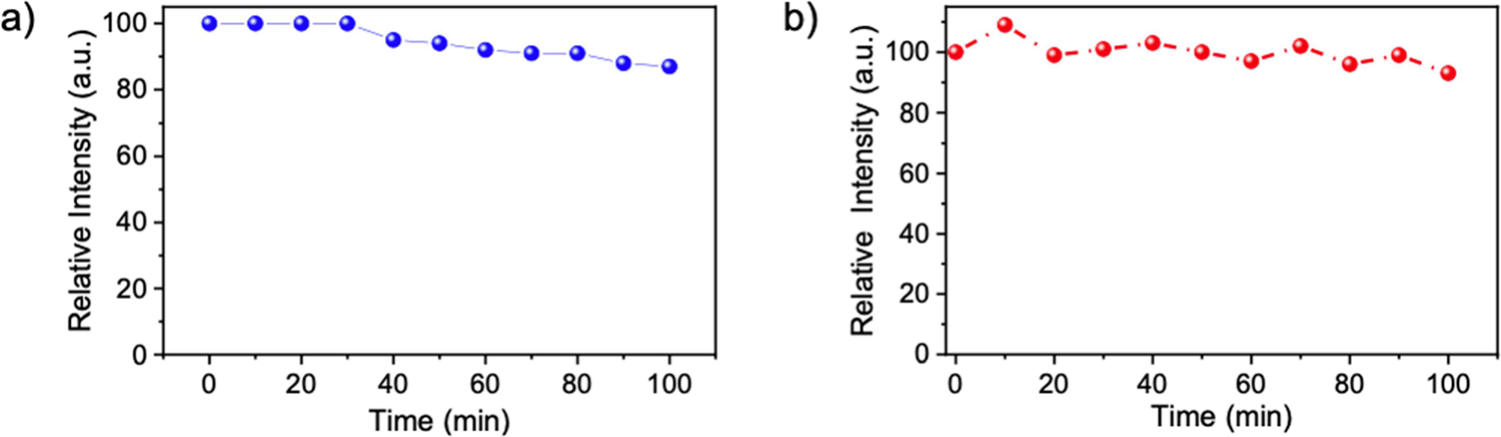
(a) Effect of UV irradiation on the stability of PL emission spectrum in aqueous and (b) solid-state film form of B-CDs.

The photostability test was conducted for both aqueous and film forms of as-prepared B-CDs to evaluate their performance as they were exposed to a UV lamp (365 nm, 6 W) for 100 min at a distance of 20 cm. The photostability of the aqueous and film forms of B-CDs was compared by measuring the changes in PL intensity over time, allowing for a direct assessment of their relative stability under UV irradiation. The relative PL intensity of the B-CDs in the aqueous form maintained its stability above 85%, while the film form exhibited stability above 93%, as illustrated in Figure 6a,b, respectively. The film form showed more excellent stability, likely due to the enhanced protection provided by the PVP matrix, which reduces degradation and environmental exposure compared to the aqueous form. The PL intensity as a function of wavelength for both aqueous and film forms of B-CDs is presented in Figure S4a,b, respectively.

In line with these findings, other studies have shown that embedding CDs in polymer matrices using a vacuum heating technique prevents fluorescence quenching and enhances their optical properties and overall efficiency.^40−42^

**Figure 7:**
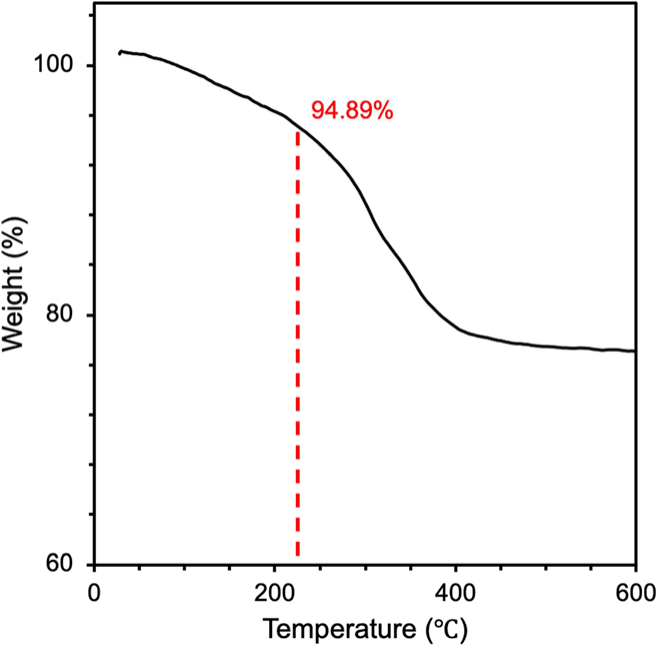
Thermogravimetry curve of powder B-CDs (heating rate of 10 °C min^–1^, nitrogen atmosphere).

Moreover, the thermal stability of powder B-CDs was tested, and the results showed that when the temperature continued to rise to 230 °C, the weight of B-CDs could still retain 94.89%, demonstrating excellent thermal stability as given in Figure 7. These results support the high potential of powder-based B-CDs as effective color conversion materials for LEDs.

## Conclusions

In this study, CDs were synthesized using a simple, fast, and easy approach, differing from traditional synthesis methods. This process was carried out under a nitrogen gas atmosphere within 1 h, with the precursor materials added directly, without using any solvent. Additionally, CDs were doped with boric acid to enhance their properties. The synthesized B-CDs possessed excellent water solubility that enabled their incorporation into the film for color-converting LEDs using a water-soluble polymer without toxic solvents, thus securing nontoxic material manufacturing during the synthesis process and the preparation of the films. Further, elemental analysis confirmed the composition of the B-CDs. At the same time, their optical properties were thoroughly investigated, highlighting an overall PLQY with only an 18% decrease when transitioning from aqueous form to film form. It produced a green emission color in a mixture with PVP at different concentrations, with CIE coordinates ranging from (0.28, 0.27) to (0.32, 0.47). This locates the synthesized B-CDs within the desired color range for color-conversion LED applications. Photostability testing showed that the aqueous form of B-CDs retained over 85% PL intensity. In comparison, the film form maintained over 93% PL intensity, likely due to the protective effect of the solid matrix of PVP. Furthermore, the thermal stability test showed that even at synthesizing temperature, B-CDs retain a mass of 94.89%, showing excellent thermal stability-one of the prime characteristics to be used in high-performance LEDs. The relationship between concentration and PLQY demonstrates the potential for tuning the optical properties of B-CDs for various applications, such as in fluorescence-based sensors or light-emitting devices.

## Supporting Information Available

EDX analysis of CDs and B-CDs, SEM images of both CDs and B-CDs, TRPL spectra of undoped CDs, and PL emission spectra of aqueous and film forms of B-CDs under UV irradiation over time (PDF)
