# Supplementary material for: Vacuum-Assisted Synthesis of Solid-State Fluorescent Carbon Quantum Dots for Color Conversion LEDs
Source: ACS Omega. 2025 Apr 11;10(15):15654–62. doi: 10.1021/acsomega.5c01047 (PMC12019725; doi:10.1021/acsomega.5c01047)
Supplement: Supplementary file 1 — ao5c01047_si_001.pdf [file ao5c01047_si_001.pdf]

Supporting Information

Vacuum-Assisted Synthesis of Solid-State Fluorescent Carbon Quantum Dots for Color Conversion LEDs

Hikmet Altintas[1], Kevser Sahin Tiras[1]*

[1] Department of Physics, Faculty of Science, Erciyes University, Kayseri 38030, Türkiye

*** Correspondence:** kevsersahintiras@erciyes.edu.tr

a) b)

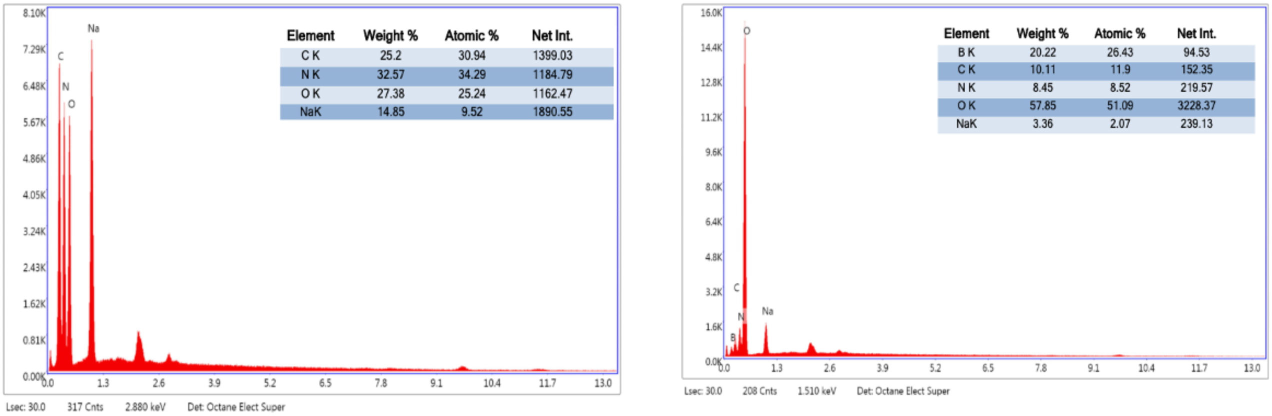

FigureS1. Energy-dispersive X-ray analysis of prepared **a)** carbon dots and **b)** boron-doped carbon dots.

a) b)

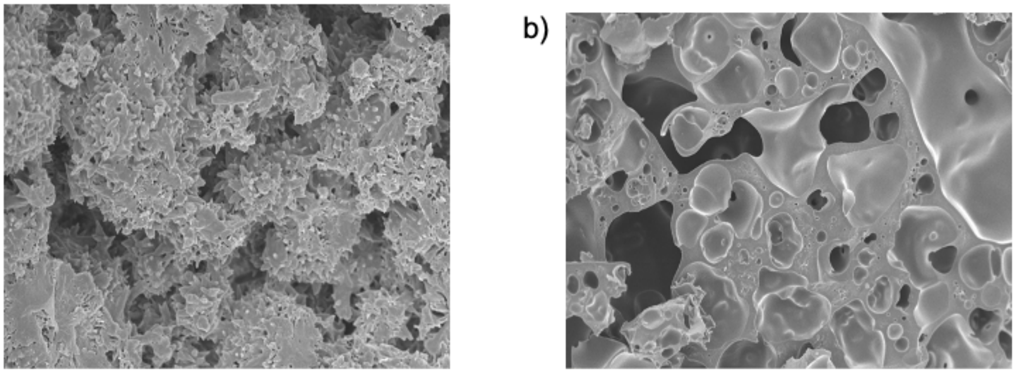

Figure S2. SEM images of **a)** carbon dots and **b)** boron-doped carbon dots.

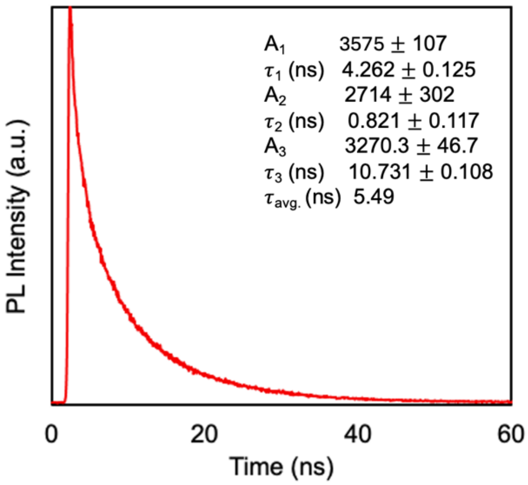

Figure S3. Time-resolved photoluminescence (TRPL) data for undoped-carbon dots.

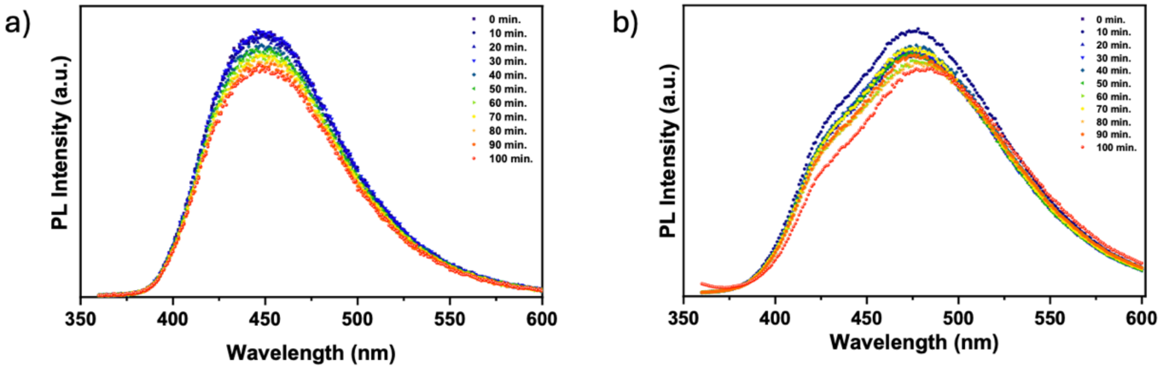

Figure S4. The effect of UV irradiation on PL emission spectrum of a) aqueous and b) film forms of B-CDs.
